# The implications of alternative pre-mRNA splicing in cell signal transduction

**DOI:** 10.1038/s12276-023-00981-7

**Published:** 2023-04-03

**Authors:** Sunkyung Choi, Namjoon Cho, Kee K. Kim

**Affiliations:** Department of Biochemistry, College of Natural Sciences, Chungnam National University, Daejeon, 34134 Republic of Korea

**Keywords:** Alternative splicing, Extracellular signalling molecules

## Abstract

Cells produce multiple mRNAs through alternative splicing, which ensures proteome diversity. Because most human genes undergo alternative splicing, key components of signal transduction pathways are no exception. Cells regulate various signal transduction pathways, including those associated with cell proliferation, development, differentiation, migration, and apoptosis. Since proteins produced through alternative splicing can exhibit diverse biological functions, splicing regulatory mechanisms affect all signal transduction pathways. Studies have demonstrated that proteins generated by the selective combination of exons encoding important domains can enhance or attenuate signal transduction and can stably and precisely regulate various signal transduction pathways. However, aberrant splicing regulation via genetic mutation or abnormal expression of splicing factors negatively affects signal transduction pathways and is associated with the onset and progression of various diseases, including cancer. In this review, we describe the effects of alternative splicing regulation on major signal transduction pathways and highlight the significance of alternative splicing.

## Molecular genetics: RNA splicing in health and disease

Cell signaling processes are affected by the varying ways that sections of messenger RNA (mRNA), the molecule that carries genetic instructions copied from a gene, are spliced together to generate several different proteins from a single gene. Kee K. Kim and colleagues at Chungnam National University in Daejon, South Korea, review the significance for cell signaling of the alternative splicing patterns of mRNAs. Mutations that lead to the splicing processes going awry are implicated in a range of diseases, including cancer. The authors examine the most recent research insights gained by applying emerging methods of genetic analysis to the role of mRNA splicing in several specific cell signaling pathways vital for normal development and health. They suggest that increasing understanding of faulty splicing in disease could open avenues towards new forms of treatment.

## Introduction

Splicing is a process in which introns, noncoding regions, are removed from a precursor messenger RNA transcript (pre-mRNA) and exons, coding regions, are joined to generate mature mRNA. The process of selective recombination of exons by site-selective splicing of pre-mRNA is called alternative splicing. Since alternative splicing allows a single gene to produce multiple mRNAs, it is a key mechanism conferring intracellular proteome diversity. Since 95% of human genes with multiple exons undergo alternative splicing, most genes encoding major signal transduction pathway components undergo alternative splicing regulation^1^. Signal transduction is the process by which a specific environmental stimulus is converted into a biochemical signal that is transferred inside a cell and the subsequent translation of the signal that leads to a change in gene expression^2^. Cells rely on various signal transduction pathways to perform functions, such as proper growth, development, differentiation, migration, and apoptosis. Because proteins produced through alternative splicing can be expressed at different levels and be involved in various biological functions, splicing regulation generally affects signal transduction pathways. For example, through the selective combination of exons encoding important domains, the protein products may interact with various other proteins with differing binding affinities^3^. Splicing-generated isoforms may change a post-translational modification (PTM) or the protein localization, thereby exhibiting unique functions^4,5^. Additionally, these isoforms may affect transcriptional or enzymatic activity^6^. Signal transduction pathway components interact with each other to form a network and transmit signals downstream. Therefore, changes in protein localization, activity, interaction, or PTM can change the cellular effect mediated by the signal transduction pathway. As a result of alternative splicing regulation, unstable proteins with reduced expression levels can be generated, and prematurely terminated truncated proteins can be generated due to frameshifting^7^. Signal transduction can be activated or inhibited by various splicing isoforms. Additionally, aberrant splicing caused by mutations or abnormal expression of splicing factors may change the behavior of a signal transduction pathway and is thus associated with the onset and progression of various diseases, including cancer. In this review, we describe the effects of various alternative splicing outcomes on key signal transduction pathways. Therefore, this review highlights the importance of alternative splicing in fine-tuning intracellular signal transduction pathway effects.

## Regulatory mechanism of alternative splicing

**Fig. 1:**
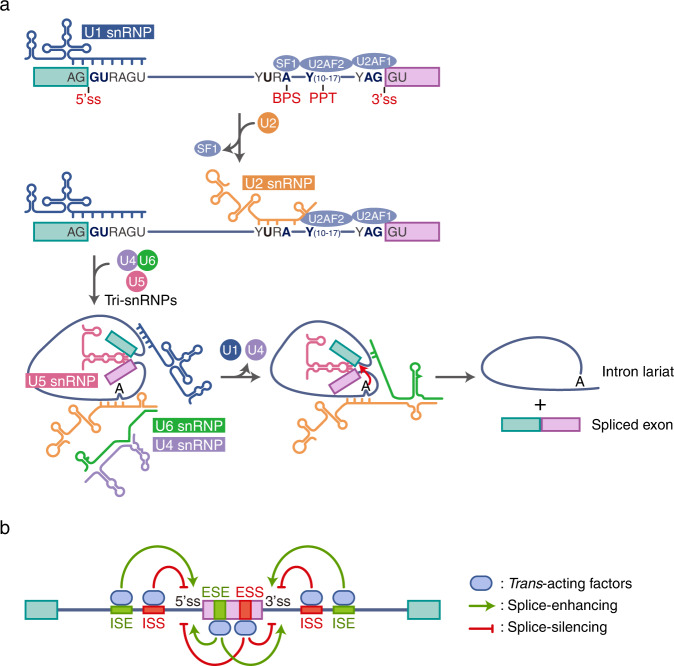
Mechanism of pre-mRNA splicing. **a** The spliceosome machinery recognizes and binds the 5’ splice site (5’ss; GU), 3’ splice site (3’ss; AG), branch point site (BPS), and polypyrimidine tract (PPT). U1 snRNP generates base pairs with the 5’ss, and U2 snRNP base pairs with the BPS. Then, tri-snRNPs consisting of U4, U5, and U6 are recruited, and U1 and U4 snRNPs are released. This step induces conformational changes in the pre-mRNA and catalyzes two successive transesterification reactions to generate ligated exon and intron lariats. **b** Exonic and intronic splicing enhancers (ESEs and ISEs, respectively) are motif sequences that enhance splicing, and exonic and intronic splicing silencers (ESSs and ISSs) are motif sequences that silence splicing. *Trans*-acting factors bind to these *cis*-regulatory sequences to regulate alternative splicing.

Splicing is regulated by the spliceosome, a large ribonucleic acid protein complex. The spliceosome components are U-enriched small nuclear RNA (snRNA) and small nuclear ribonucleoprotein particles (snRNPs), which are composed of several nuclear proteins^8,9^. snRNPs are classified according to their association with snRNAs; U1, U2, U4, U5, and U6 are representative snRNPs. Splicing is controlled by regulatory consensus sequences on pre-mRNA that define exon/intron boundaries^10^; these sequences include a 5′ splice donor site (5′ss), branch point site (BPS), polypyrimidine tract (PPT), and a 3′ splice acceptor site (3′ss) (Fig. 1a). U1 snRNA binds to the GU dinucleotide of the 5’ss of a pre-mRNA via a base-pair interaction. The splicing factor SF1 recognizes and binds to a BPS. In contrast, U2AF is a heterodimer composed of U2AF2 and U2AF1, which promotes the replacement of SF1 with a U2 snRNP in the BPS after binding to the PPT and an AG dinucleotide sequences in the 3’s, respectively. Then, the tri-snRNP complex containing the U4, U5, and U6 complexes is recruited. The binding of U4/U5/U6 tri-snRNPs to mRNA induces a conformational rearrangement, and U1 and U4 are then released. This sequence change induces the activation of two transesterification reaction steps, triggering 5’ss cleavage and catalyzing 3’ss and exon ligation. Finally, the mRNA is generated with introns cleaved, and the snRNPs are released from the spliceosome complex and recycled for consumption in additional splicing rounds^11–13^.

Additionally, *cis*-regulatory sequences in exons and introns increase the fidelity of the splicing process (Fig. 1b). *Cis*-regulatory sequences include exonic and intronic splicing enhancers (ESEs and ISEs, respectively) or exonic and intronic splicing silencers (ESSs and ISSs, respectively)^14^. Exons and introns are recognized based on the presence and interaction of *cis*-regulatory sequences and *trans*-acting factors, such as RNA-binding proteins. *Cis*-regulatory sequences modulate alternative splicing by either activating or inhibiting the occupation of nearby splice sites by recruiting *trans*-acting factors to enhancer or silencer sites. Arginine/serine dipeptide-rich (SR) proteins, *trans*-acting factors, recruit U1 snRNP, or U2AF to a 5’ss and BPS at an RNA recognition motif, forming the nascent spliceosome E complex and promoting bound exon incorporation^15–18^. In contrast to SR proteins, which are splicing enhancers, nuclear heterogeneous RNPs (hnRNPs) bind to an ISS and inhibit exon inclusion^19,20^. However, various *trans*-acting factors that induce exon inclusion and exclusion may exert opposite effects depending on specific site to which it binds^21,22^. These *trans*-acting factors block snRNP recruitment or access to consensus sequences and induce structural changes in RNA, thereby affecting splice site selection. They further fine-tune alternative splicing through strong cooperative and competitive effects^23–25^.

**Fig. 2:**
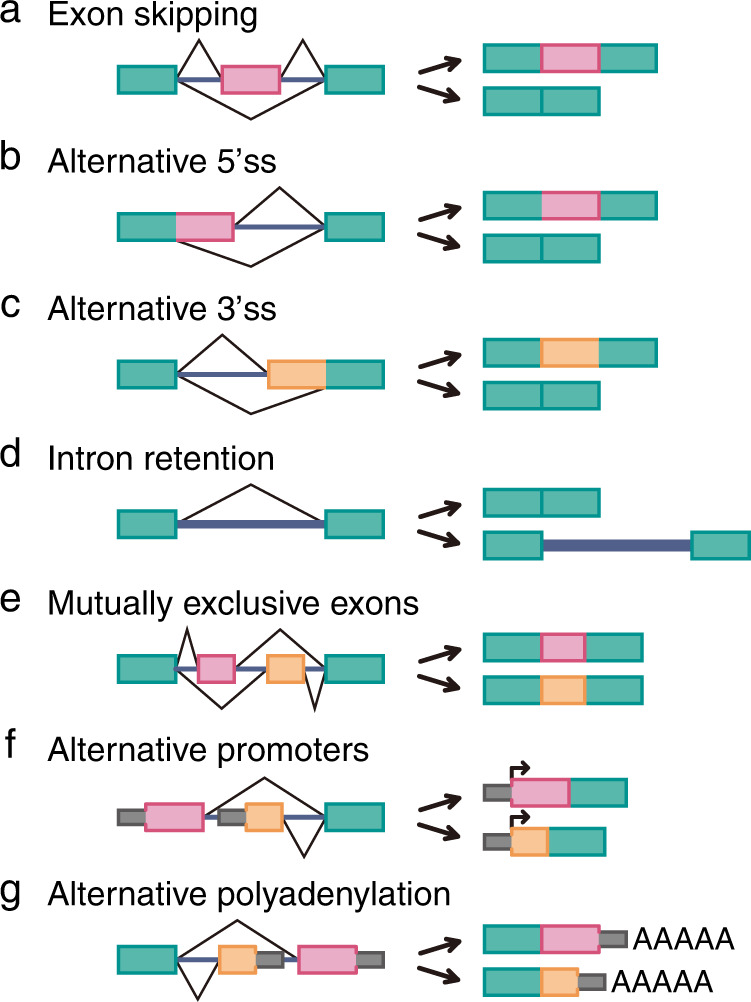
Types of alternative splicing. Seven main types of alternative splicing events are depicted. Exons are represented by boxes, and introns are represented by lines. Constitutive exons are green, and alternatively spliced exons are pink and orange. Bent lines indicate an alternative splicing event. Promoters (P) are indicated with arrows and polyadenylation sites with AAAAA. Exon skipping (**a**), alternative 5’ss (**b**), alternative 3’ss (**c**), intron retention (**d**), mutually exclusive exons (**e**), alternative promoter (**f**), and alternative polyadenylation (**g**) are shown.

Systematic analysis using next-generation sequencing and computational techniques has thus far revealed seven basic types of alternative splicing. Among alternative splicing events, exon skipping is the most frequent alternative splicing type in higher eukaryotes, and the exon that is skipped is called a cassette exon (Fig. 2a). The next most common alternative splicing type is called an alternative 5’ss or 3’ss event, in which the length of an exon with two or more splice sites is changed (Fig. 2b, c). Introns can be retained in mature mRNA, which is a common alternative splicing outcome in plants and lower metazoans (Fig. 2d)^26^. A mutually exclusive exon is generated when one exon of two consecutive alternative exons is included when the other exon is excluded (Fig. 2e). Finally, alternatively spliced transcripts are generated by alternative promoters or alternative polyadenylation involving more than one initiator or terminator exon (Fig. 2f, g). Key signal transduction pathway components are subjected to these different alternative splicing regulatory mechanisms. The effects of alternative splicing on each of the major signal transduction pathways are described below.

## Hedgehog pathway

**Fig. 3:**
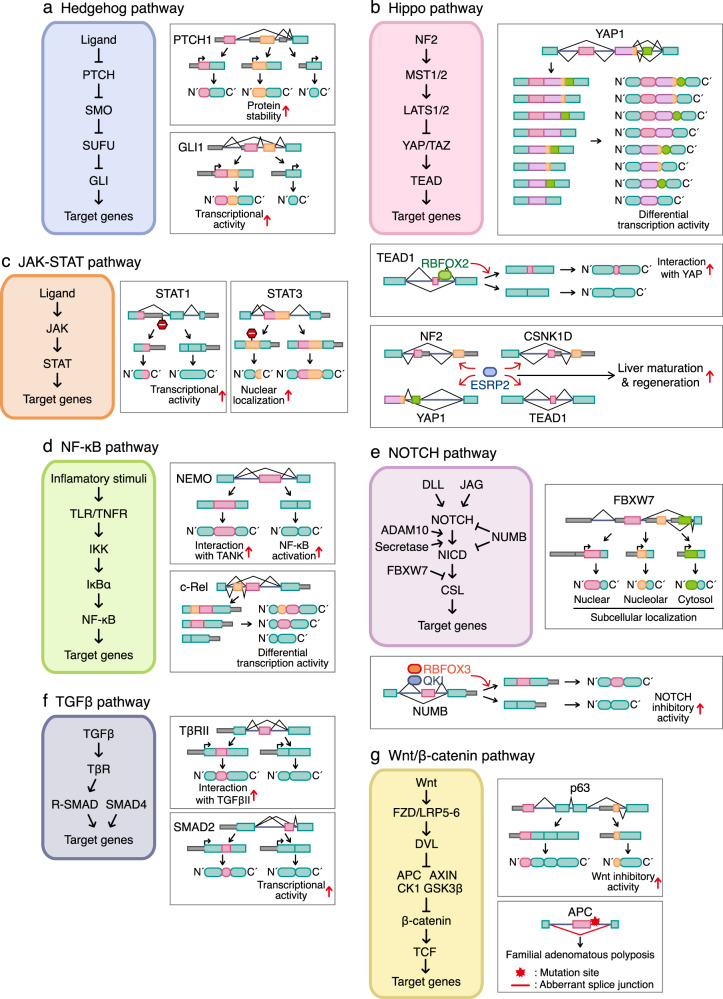
Differential activities of splicing isoforms in cell signal transduction. Several genes that are involved in signal transduction produce alternative splicing-derived proteins with differential activities in regulating signaling pathways. A brief mechanism of intracellular signal transduction and representative alternative splicing events that regulate them are presented. The seven major signal transduction pathways include the Hedgehog pathway (**a**), Hippo pathway (**b**), JAK-STAT pathway (**c**), NF-κB pathway (**d**), NOTCH pathway (**e**), TGF-β pathway (**f**), and Wnt/β-catenin pathway (**g**).

The hedgehog pathway is essential for normal embryonic patterning and development and plays an important role in the maintenance, regeneration, and homeostasis of adult tissues^27,28^. In the absence of the hedgehog ligand, Ptch (protein patched homolog) is located in primary cilia, sequestering the downstream transducer SMO (smoothened homolog) into intracellular compartments and inhibiting SMO activity^27–34^. The transcription factor GLI is sequestered in the cytoplasm by SUFU and phosphorylated by protein kinase A, CK1 (casein kinase 1), and GSK3β (glycogen synthase kinase 3β). Phosphorylated GLI is processed by the proteasome into its repressor forms (GLI2R and GLI3R). Therefore, Hh pathway target gene expression is turned off. When the hedgehog ligand is present, Ptch leaves cilia and does not inhibit SMO, allowing it to accumulate in the primary ciliary membrane. SMO is then activated, mitigating the inhibitory effect of the cytoplasmic sequestrated factor SUFU and leading to GLI activation. Activated GLI migrates to the nucleus to activate the transcription of Hedgehog pathway target genes (Fig. 3a).

Because of their alternative promoter, PTCH1 isoforms with different first exons show higher protein stability than wild-type PTCH1 and thus inhibited SMO, GLI1, and GLI2 to a greater extent^35–39^. Thus, isoforms with different N-terminal tails induce various levels of Hedgehog signaling inhibition. Additionally, tissue-specific alternative splicing and nevoid basal cell carcinoma syndrome (NBCCS)-related abnormal PTCH1 splicing, which includes exons 1-5, exon 10, and the novel exon 12b, were detected using exon junction microarrays^40^. A PTCH2 variant lacking exons 9 and 10, which are in the conserved sterol-sensing domain, and a PTCH2 variant lacking the final exon, exon 22, have been reported^41^. Of these two variants, only the isoform in which PTCH2 exon 22 was skipped inhibited hedgehog activity. Both isoforms failed to inhibit the activated form of SMO but they translocated SMO that had been dispersed in the cytoplasm to the juxtanuclear region.

Longer transcripts (α and β in mice and β in humans) and shorter transcripts (γ) are generated according to the number of noncoding exons in the GLI1 5’ UTR^35,42^. The longest variants were expressed in normal tissues but not in BCC (basal cell carcinoma) or human keratinocyte cell lines, whereas GLI-γ was expressed in BCC and HaCaT cells. Additionally, GLI-γ expression was induced by TPA (12-O-tetradecanoylphorbol 13-acetate) treatment in mouse skin, but the levels of the longer variants were reduced. These observations suggest an association between GLI-γ with actively proliferating keratinocytes and GLI-α and GLI-β expression and quiescent cells, demonstrating a clear correlation with proliferative status. Moreover, an increased 5’-UTR length has been confirmed to reduce the translation efficiency of GLI1 mRNA, and differential inclusion of the first exon has been shown to depend on SNPs (rs10783826, rs10783827, and rs118093490)^43^. GLI1-γ expression after 5’ noncoding exon skipping may be an important oncogenic contributor. GLI1 variants encoded a truncated GLI1 (GLI1ΔN) protein, which lacked the N-terminal SUFU-binding domain because exons 2 and 3 were skipped^44^. In adult human tissues, GLI1ΔN mRNA was expressed with wild-type GLI1 at an approximate 1:1 ratio, but a generally lower and variable expression pattern of GLI1ΔN mRNA was observed in tumor cell lines. GLI1ΔN weakly activated transcription; however, in specific cellular contexts, GLI1ΔN exerted a more potent transcription-inducing effect than full-length GLI1. A truncated GLI1 splice variant (tGLI1) in which all of exon 3 and part of exon 4 were skipped, was not expressed in normal cells but was highly expressed in glioblastoma multiforme (GBM) and other cancer cells^45^. In contrast to wild-type GLI1, tGLI1 specifically targeted CD24, an invasion-associated gene, inducing its transcription and was involved in increased tumor aggressiveness by promoting GBM cell motility and invasiveness.

Various GLI2 variants have been reported, and the isoform in which GLI2 exon 3 is skipped induced reporter gene activation similar to the full-length isoform^46,47^. In contrast, the isoform in which GLI2 exons 4 and 5 were skipped was expressed at relatively low protein levels but induced 10-fold greater reporter gene activation. The expression levels of GLI2∆N (GLI2 with the N-terminus truncated) and GLI2∆C (with the C-terminus truncated) were increased by TGF-β, and these isoforms were expressed mainly in cells with a highly migratory and invasive phenotype^48^. Mutations (c.473 + 5 G > A and c.473 + 3 A > T) leading to GLI3 exon 4 skipping and lack the most functionally important domains and have been identified in patients with polydactyly^49,50^.

## Hippo pathway

The Hippo pathway is an evolutionarily conserved signaling pathway that regulates many biological processes, including cell growth, survival, metastasis, and organ size and regeneration^51,52^. Thus, Hippo pathway dysregulation leads to abnormal cell growth and the development of tumors^53^. This pathway is turned on and off by various internal and external signals, such as those related to mechanotransduction, cell‒cell contact inhibition, cell adhesion, cell polarity, hormone signaling, and extracellular matrix (ECM) stiffness^52,53^. In soft-ECM, small-surface, and high-cell-density conditions, the Hippo pathway phosphorylated MST1/2 and activated LATS1/2 to phosphorylate YAP/TAZ. Phosphorylated YAP/TAZ interact with 14-3-3 proteins and cause their cytoplasmic retention^54,55^. Further YAP/TAZ phosphorylation by casein kinase 1 induced β-TrCP-mediated ubiquitination and proteasomal degradation^56–58^. In contrast, under conditions such as a stiff ECM, large surface areas, sparse cells, and stretched cells, the Hippo pathway is turned off, and YAP/TAZ was not phosphorylated and was translocated to the nucleus. YAP/TAZ bound to TEAD, a transcription factor, and induced the transcription of genes important for cell proliferation, survival, and migration (Fig. 3b).

The human YAP1 gene comprises nine exons, including exons 4 and 6, which are cassette exons, and exon 5, which carries a 5’ss. Using these exon combinations, researchers identified eight transcripts encoding an alternatively spliced YAP1 isoform^59^. The YAP1 isoform with exon 5 extended, exon 6, or both was predicted to disrupt the leucine zipper region in terms of protein structure and to affect various protein‒protein interactions. The second YAP WW domain is encoded by exon 4, and TEAD-mediated transcriptional activity is reduced when exon 4 is skipped^60^. In particular, the leucine zipper disruption by the extended exon 5 led to a greater decrease in transcriptional activity than the absence of the second WW domain.

The SR protein splicing factor SRSF1, a direct target of transcription factor and oncoprotein MYC, promotes TEAD1 exon 5 inclusion^61^. The TEAD1 full-length isoform showed greater transcriptional activity and oncogenic properties than the TEAD1 isoform without exon 6^3^. Exon 6 is the region coding for the DNA binding domain of TEAD1, but there was no difference in the DNA binding activity of the two isoforms, and rather, it was confirmed that the interaction with YAP was affected. Through a TCGA database analysis, the Hippo target gene expression level did not correlate with the expression level of TEAD1 itself but showed a positive correlation with the percent spliced in (PSI) value of exon 6, which comprises only 12 nucleotides (nt). In addition, patients with high TEAD1 exon 6 levels exhibited a lower survival rate than those not carrying TEAD1 exon 6. TEAD1 exon 6 is harbored by RBFOX2, and it has been confirmed that RBFOX2 affects Hippo target gene expression because of TEAD1 splicing changes.

TEAD4 variants with exon 3 skipped by RBM4 are translated starting at exon 6, yielding a short TEAD4 protein isoform (TEAD4-S)^62^. TEAD4-S does not carry an N-terminal DNA-binding domain but harbors a C-terminal YAP-binding motif. In contrast to full-length TEAD4 (TEAD4-FL), which predominantly localizes to the nucleus, TEAD4-S is located in both the nucleus and cytoplasm and exerts a dominant-negative effect on YAP activity by antagonizing TEAD4-FL. TEAD4-S expression was decreased in cancer cells, and TEAD4-S inhibited cancer cell proliferation and the epithelial-mesenchymal transition (EMT). Consistent with this, patients with high TEAD4-S scores showed longer survival.

ESRP2 (epithelial splicing regulatory protein 2) downregulation reactivated the neonatal splicing program and induced the exclusion of alternative Nf2, Csnk1d, Yap1, and Tead1 exons, which are involved in the Hippo pathway^63^. ESRP2 has been shown to modulate hepatocyte proliferation in response to chronic liver injury by attenuating Hippo pathway activation and activating gene expression associated with cell proliferation. Similarly, TNF-α (tumor necrosis factor-α) and IL-1β (interleukin-1β), proinflammatory cytokines that accumulate in alcohol-damaged livers, have been shown to inhibit ESRP2 in adult hepatocytes^64^. ESRP2 loss increased YAP/TAZ activity by regulating the splicing of CSNK1D and NF2, thereby upregulating Hippo target genes^64,65^. ESRP2 inhibition increased the viability and regenerative capacity of hepatocytes, increasing their survival. These results revealed the cause of liver failure in patients with SAH (severe alcoholic hepatitis) from the perspective of an ESRP2-dependent RNA-splicing program.

## JAK-STAT pathway

More than 50 cytokines and growth factors, such as hormones, interferons (IFNs), and interleukins (ILs), transmit signals downstream through the JAK (Janus kinase)-STAT (signal transducer of activators of transcription) pathway to regulate hematopoiesis, immune responses, pathogen resistance, cell growth and survival, tissue repair, apoptosis, adipogenesis, and cell differentiation^66–69^. When a cytokine binds to a specific receptor, the receptor multimerizes, bringing JAK into close physical proximity. JAK then mediates transphosphorylation of tyrosine residues to create a STAT-docking site that binds to the cytoplasmic domain of the receptor. STATs are in turn phosphorylated and activated, driving their dimerization. STAT-STAT dimers are translocated to the nucleus, where they bind directly to DNA and upregulate the expression of related genes (Fig. 3c).

JAK2 exon 14 skipping has been found in patients with primary myelofibrosis (PMF) and myeloproliferative neoplasms (MPNs)^70,71^. A somatic guanine-to-thymine substitution (c.1849G > T) located in the terminal part of JAK2 exon 14 was observed in MPN patients, resulting in changes in amino acids (V617F), altering the pseudokinase domain structure. JAK2-V617F has been observed in most patients with polycythemia vera and approximately 60% of patients with PMF. JAK2 exon 14 skipping was a more frequent event in patients with the JAK2-V617F mutation. The truncated JAK2 isoform generated by JAK2 exon 14 skipping dimerized with wild-type JAK2 to activate the kinase domain and possibly triggered JAK2-STAT pathway activation, suggesting that it may play an important role in MPN pathophysiology^70^. However, the hypothesis suggesting that the truncated protein isoform of JAK2 may exert an antiproliferative effect has also been proposed^71^; therefore, further research is needed to determine truncated JAK2 function. Additionally, a JAK3 splice variant with a different C-terminus was isolated from breast (B) spleen (S) tissue and activated monocytes (M)^72^. JAK3-S and M encode amino acids 1068 (alanine) and 1069 (glutamine) as a result of differential splicing in which GCTGAG is the 5’ss, whereas JAK3-B generates a read-through transcript without accessing the 5’ss. JAK3-S has been detected mainly in hematopoietic cell lines, whereas JAK3-B and M have been detected in cell lines derived from a broad array of hematopoietic and epithelial tissues and showed a wider expression profile. JAK3-B did not show kinase activity because subdomain XI in the tyrosine kinase core was absent. Competition between kinase-active JAK3 and defective JAK3B attenuated downstream responses, suggesting that JAK3B is a dominant-negative isoform that transactivates JAK3 signaling. Additionally, these three JAK3 isoforms recruit different proteins and substrates within a cell, conferring signal transduction complexity. The mutation (c.2652 C > T; pV884V) in JAK3 exon 19, which has been identified in patients with severe combined immunodeficiency (SCID), did not encode an altered the amino acid sequence but created a new 5’ss^73^. As a result, 29 nt were deleted at the 3’ end of exon 19, resulting in a premature stop codon. A protein encoded by this variant was not detected, indicating that it was either not expressed or was rapidly degraded. Taken together, these studies suggest that JAK3 exons 17-23 encode a kinase domain and that splicing events involving these exons regulate the related C-terminal region and are very important for altering JAK3 function.

In addition to the full-length α-isoforms of STAT1, 3, 4, and 5, evolutionarily conserved β-isoforms carry truncated C-terminal transactivation domains (TADs)^74,75^. Initially, β-isoforms were considered to be dominant-negative variants, but some studies have reported overlapping or unique functions of α-isoforms^76–78^. STAT1β was found to be transcriptionally active at lower levels than STAT1α in response to IFN-γ signaling, and STAT1α and STAT1β affected the transcription of distinct sets of genes^79,80^. In experiments with knock-in mice, NK cells from Stat1β/β mice showed impaired maturation and effector functions, although the effects were less severe than those in NK cells from mice lacking STAT1 (Stat1 −/−)^81^. This study confirmed that NK cell function was more efficiently affected by STAT1α. Additionally, normal phosphorylation and DNA binding did not occur after STAT1 exon 3 skipping induced by the G372C mutation, suggesting that abnormal signal transduction was induced in response to IFN-γ and IFN-α, which are related to the susceptibility to intracellular pathogens and viruses^82^. An alternative 3’ss with a difference in STAT3 exon 23 at position 50 nt produced STAT3-α and a frame-shifted prematurely terminated STAT3-β isoform^83^. STAT3-α regulates cellular responses to IL-6 and mediates IL-10 function in macrophages. In vivo, STAT3-β rescued the embryonic lethality induced by STAT3-null mutations and led to the expression of specific STAT3 target genes. STAT3-β is more efficiently transported to the nucleus, can stay in the nucleus longer than STAT3α, and regulates a greater number of genes^84^. A splicing variant of the GTAGTT tandem 5’ss in STAT3 exon 21 has been identified^85^. These isoforms, with or without Ser-701 inclusion, were required for optimal STAT3 function in ABC DLBCL (activated B-cell-like diffuse large B-cell lymphoma) cells^86^. Stat4α was required for the maximal induction of IL-12-induced IFN-γ production, whereas Stat4β was required for IL-12-stimulated proliferative responses^87^. Additionally, certain genes were regulated in common, but other target genes were specifically regulated by the STAT4 isoform.

## NF-κB pathway

The nuclear factor kappa B (NF-κB) family of transcription factors regulates the expression of various genes that mediate many aspects of innate and adaptive immunity, inflammation, proliferation, cell death, angiogenesis, EMT, and tumor cell invasion and metastasis^88–94^. In the NF-κB signaling pathway, TNF-α, LPS (lipopolysaccharides), and IL-1 activate TNFRs (TNF receptor), TLRs (Toll-like receptors), and IL-1R (IL-1 receptor), respectively. Integral membrane receptors activate the IKK (IκB kinase) complex. The IKK complex is composed of a heterodimer comprising catalytic IKKα and IKKβ subunits and NEMO (NF-κB essential modulator). Activated IKKβ in the IKK complex phosphorylates serine residues 32 and 36 in human IκBα, leading to the ubiquitination and proteasomal degradation of IκBα. When IκBα is degraded, NF-κB homo or heterodimers are activated by PTM and translocated to the nucleus, where they bind to DNA consensus sequences and activate the transcription of target genes (Fig. 3d).

A variant of TNFRSF1B, encoding TNFR type 2, icp75TNFR carries a 5’-UTR and an alternative first exon^95^. Wild-type TNFRSF1B was found on the cell surface, but due to the alternative first exon not carrying a signal peptide, the icp75TNFR isoform was diffused throughout the cell and colocalized with some mitochondria. icp75TNFR bound to TNF, which resulted in NF-κB activation in TNF-stimulated cells. A variant in which human TLR1 exon 2 was skipped and five variants generated by two alternative 3’ss and three 5’ss of TLR2 exon 2 produced identical proteins^96,97^. However, the 5’-UTR difference may have affected mRNA stability and translation.

The isoform caused by exon 5 skipping in NEMO, the noncatalytic subunit of IKK, efficiently mediated IKK and NF-κB activation but did not mediate IKK activation induced by HTLV-1 (T-cell leukemia virus type-1) Tax^98^. A mutation in the 5’ss of exon 6 (IVS6 + 5 G → A(1027 + 5 G → A)) identified in a family with severe immunodeficiency induced skipping of NEMO exons 4, 5, and 6, resulting in a small protein lacking part of the coiled-coil motif (CC) 1 and the linker between CC1 and CC2^99^. This mutation resulted in low levels of IκBα degradation and impaired NF-κB signaling.

NFKBIZ, the gene encoding IκBζ, generates 15 alternative splicing variants, but only 3 of these mRNAs encode proteins^100^. A long IκBζ(L) variant consisting of 14 exons was induced by LPS stimulation^101^. The short IκBζ(S) variant lacks exon 3, where the initiation codon is located, and therefore lacks 99 amino acids in the N-terminus. As a result of alternative splicing at exon 7, the central part of the transactivating domain (TAD), the IκB-ζ(D) variant with the shortened exon 7, was induced upon LPS stimulation, similar to IκBζ(L)^102^. IκB-ζ(D) showed NF-κB inhibitory activity but no transactivation activity due to the TAD deletion.

The NF-κB family comprises five genes: NF-κB1, NF-κB2, relA, c-rel, and relB. Multiple splicing variants of relA, relB, and NF-κB2 have been identified in the lungs of CD14 knock-out mice after burn injury^103^. Most variants encode proteins that include frameshifts, are prematurely terminated or lack domains essential for NF-κB transcription factor function. Thus, these splicing changes could serve as one of the mechanisms regulating excess NF-κB activity in response to stress signals, such as burns.

Two NFKB1 SNPs (rs230511 and rs230504), which induced exon 4 and 5 skipping and produced an out-of-frame NFKB1 mRNA, have been identified in the Aymara people from the Andean highlands^104^. These two SNPs correlated with higher hemoglobin levels and lower white blood cell counts. Since truncated nonfunctional NF-κB inhibits inflammation, the NFKB1 haplotype variant is thought to be the main explanation for the adaptation of the Aymara population to high altitudes. Additionally, numerous NFKB1 mutations in patients with common variable immunodeficiency (CVID) or related diseases are located at the splice site and produce abnormally spliced mRNA variant^105^. c-Rel with an Alu sequence inserted between exons 8 and 9 generates a 32 amino acid longer variant and a variant with exon 9 skipping^106^. Both c-Rel isoforms showed enhanced DNA binding and transactivation compared with these functions in wild-type c-Rel.

## NOTCH pathway

The Notch pathway is an evolutionarily conserved signal transduction pathway that regulates embryonic development, cell proliferation, differentiation, and death; determines cell fate; and maintains adult tissue homeostasis. Mammals carry Notch family members (Notch-1, -2, -3, -4), which act as receptors, and Delta-like ligands (DLL1, DLL3, and DLL4) and Jagged ligands (JAG1, JAG2), which are ligands in the Notch pathway^107^. Notch receptors are produced in the endoplasmic reticulum and transported to the plasma membrane. The interaction of a Notch extracellular domain with a cognate ligand result in a cascade of receptor cleavage reactions mediated by ADAM10 and secretase. After cleavage, the Notch intracellular domain is released into the cytoplasm, migrates to the nucleus, and binds to the NOTCH pathway transcription factor CSL to activate the transcription of Notch target genes, such as Myc, p21, HES, and HEY family genes (Fig. 3e)^108^.

Aberrant splicing variants have been identified in patients with myeloid leukemia, in which exon 12 and exons 17 and 18, which encode parts of the NOTCH2 extracellular EGF-like domains, are skipped^109^. These NOTCH2 splice variants impair the function of full-length NOTCH2 and act as dominant-negative variants, blocking the signaling pathway in AML cells. Additionally, exon 12 in NOTCH2 has been suggested to be related to apoptosis^110^. A mutation in which a part of the NOTCH3 intron 3 branch point site was deleted, causing abnormal splicing, has been found in a family with late-onset cerebral autosomal dominant arteriopathy with subcortical infarcts and a leukoencephalopathy (CADASIL) phenotype, suggesting an association between this variant and the pathologically confirmed CADASIL phenotype^111^. Significantly lower expression of NOTCH3 exon 16 mRNA encoding three EGF-like domains has been observed in patients with the GCB (B-cell-like) subtype of DLBCL (diffuse large B-cell lymphoma), classified as a vincristine-resistant variant^112^. These patients exhibited low survival rates, demonstrating the potential of NOTCH3 exon 16 transcript levels to be used as prognostic and predictive biomarkers. In addition, the A-to-G transition mutation in the 3’ ss of NOTCH3 exon 4 found in CADASIL patients resulted in an in-frame deletion of 7 amino acids, including the 6th cysteine residue of the second EGF domain^113^. The unpaired cysteine residue promoted abnormal NOTCH3 oligomerization, possibly playing a key role in the prognosis of patients with CADASIL. Taken together, these studies suggest that the splicing of exons encoding EGF-like domains in extracellular domains can affect receptor-ligand interactions that play important roles in the function of NOTCH.

A JAG2 variant that lacks exon 24 has been identified in breast cancer^114^. Exon 24 encodes 984-994 amino acids included in the Von Willebrand Factor C domain, but further research is needed to determine the function of this isoform. NOTCH is ubiquitinated and degraded by FBXW7 (F-Box and WD repeat domain-containing 7). FBXW7 encodes three isoforms, α, β, and γ, which differ in the N-terminus as a result of an alternative promoter. FBXW7α is located mainly in the nucleus, β is located in the cytoplasm, and γ is located in the nucleolar region^115^. The FBXW7α mRNA expression level in cells was the highest, approximately 100-fold that of γ^116^. Although FBXW7α is predominantly expressed in most human tissues, FBXW7β mRNA is abundantly expressed in the brain and thymus and is absent or minimally expressed in other tissues, and the expression of FBXW7γ mRNA appears to be restricted to the heart and skeletal muscle^117^. There are seven FBXW7α 5′-UTR variants that produce the same protein but demonstrate differential translational efficiencies. In addition to FBXW7β reducing the stability of the NOTCH intracellular domain, Notch-regulated HES5 has been shown directly repressing FBXW7β transcription, forming a Notch/Hes5/FBXW7β positive feedback loop^118^. FBXW7β expression was increased by genotoxic stress stimuli, and p53 upregulated FBXW7β. In contrast, the expression of FBXW7α and γ was independent of p53 activity and showed a limited response to most stress stimuli.

NUMB prevents the translocation of the NOTCH intracellular domain to the nucleus. RBFOX3 plays an important role in neuronal differentiation progression during vertebrate development by inhibiting exon 12 inclusion through two UGCAUG elements present in NUMB intron 11^119^. QKI also inhibits NUMB exon 12 inclusion by recognizing two QKI-binding sequences adjacent to the 3’ splice site of intron 12 and selectively competing with SF1 for binding to the branch point site^120^. The exclusion of exon 12, which encodes 48 amino acids in the protein-protein interaction domain PRR, inhibits cell proliferation and prevents Notch pathway activation. NOVA1 excludes SORBS2 exon 3, and SORBS2 containing exon 3 inhibits NOVA1 and NOTCH1, promoting colorectal cancer cell migration mediated through the NOTCH pathway^121^.

## TGF-β pathway

The TGF-β (transforming growth factor beta) superfamily includes ligands such as TGF-β, activin/inhibins/Nodal, BMPs (bone morphogenetic proteins), GDFs (growth and differentiation factors), and AMH (anti-Müllerian hormone)^122–125^. The TGF-β pathway is involved in tissue homeostasis and plays an important role in disease development by regulating various cellular processes, including cell proliferation, differentiation, apoptosis, angiogenesis, immune responses, cell invasion, migration, EMT, and ECM production. TGF-β signaling is initiated by the binding of TGF-β to a specific set of TβRII (serine and threonine kinase receptors, type II) and TβRI (serine and threonine kinase receptors, type I) receptors on the cell membrane. TβRII binds to TGF-β and induces the formation of a heterotetrameric receptor complex that activates TβRI and *trans*-phosphorylates it. This phosphorylated receptor, in turn, phosphorylates TGF-β receptor-specific SMADs (R-SMAD: SMAD2 and SMAD3) inducing TGF-β and activin signaling and Smad1/5/8 for BMP signaling. Carboxy-terminal phosphorylation of Smads by activated receptors leads to the formation of a heterocomplex with the co-Smad Smad4 and is translocated to the nucleus, where it cooperates with transcription factors and transcriptional coactivators to regulate target gene transcription. SMAD6 and SMAD7 inhibit signaling by antagonizing R-SMAD activation. In addition to activating SMAD proteins, TGF-β signaling can transmit ERK, MAPK, SAPK/JNK, PI3K-AKT, and NF-κB signals. This canonical and noncanonical TGF-β signaling is also affected by other signaling pathways, such as the Hedgehog, Notch, RAS, TNF, interferon, and Wnt pathways (Fig. 3f).

The TGFBR1 mutation c.973 + 1 G > A has been found in patients with LDS (Loeys–Dietz syndrome), and c.806-2 A > C has been found in patients with MSSE (multiple self-healing squamous epitheliomas) ^126^. In the LDS variant, activation of a cryptic 5’ss located 9 nt upstream of the 5’ splice site in intron 5 was induced, or exon 5 was skipped, generating two functionally inactive proteins. The MSSE variant is a 3’splice site mutation in intron 4 that activates a cryptic 3’ss located 76 bp downstream to generate an out-of-frame transcript. The frequency of a G → A mutation located 24 nt downstream of TGFBR1 intron 7 was significantly increased in RCC (renal cell carcinoma) and TCC (transitional cell carcinoma) patients compared to individual controls without cancer^127^. Abnormal splicing of the exon encoding the kinase domain leads to functional alterations in the TGF-β signaling pathway and shows the potential to induce various diseases, such as LDS, MSSE, RCC, and TCC.

Insertion of a 75 nt intron between exons 1 and 2 of TGFBR2 produces the membrane-anchor isoform TβRII-B^128^. TβRII binds only to isoforms TGF-β1 and TGF-β3, whereas TβRII-B binds to TGF-β1, 2, and 3 and forms a complex with TβRI, TβRII, and TβRIII. TβRII-B transinduces TGF-β2 signaling mediated through Smad2, independent of TβRIII, and TβRII-B signaling mediated through the Smad2/3 pathway was induced mainly through TβRI activation^129^.

The exon 3-skipped isoform of Smad2, which encodes a part of MH1 (Mothers against decapentaplegic (Mad) homology domain-1) that is critical for direct DNA binding, binds to DNA containing the AP-1 (activating protein-1) site, and its transcriptional activity is higher than that of an exon 3 inclusion isoform^130,131^. Additionally, the level of the alternatively spliced variant of Smad2 lacking exon 3 was increased during early postnatal brain maturation in mice and was expressed at higher levels than the exon 3-included variant at all stages, suggesting that it has a special function during brain differentiation^132^. Smad3 exon 3 skipping generates an isoform with a partially truncated linker region that shows TGF-β-dependent transcriptional activity^133^. Smad8B is generated by the deletion of 47 amino acids, including the SSXS motif that is phosphorylated by the BMP type I receptor ALK2 in the C-terminal MH2 region of Smad8^134^. This isoform is located in the cytoplasm and is a dominant BMP signaling inhibitor.

## Wnt/β-catenin pathway

The Wnt/β-catenin pathway modulates embryonic development and adult tissue homeostasis by influencing physiological activities, such as cell proliferation, division, differentiation, migration, polarity, and apoptosis^135–139^. In the absence of the Wnt ligand, β-catenin is phosphorylated by the destruction complex, which is composed of Axin, APC (adenomatous polyposis coli), CK1α (casein kinase 1α), and GSK3β. Then, β-catenin phosphorylation leads to subsequent ubiquitin-proteasome degradation. In the Wnt-activation state, the Wnt ligand binds to a coreceptor composed of Frizzled and LRP5/6 (low-density lipoprotein receptor-related protein 5/6). The receptor LRP5/6 is phosphorylated by CK1 and GSK3β, and DVL and AXIN are then recruited, leading to GSK3β inhibition. This process induces the disassembly of the destruction complex, and β-catenin accumulates in the cytoplasm, is translocated to the nucleus, and then interacts with TCF/LEF transcription factors to induce target gene transcription (Fig. 3g).

Multiple APC splice site mutations have been identified in FAP (familial adenomatous polyposis) patients^140^. Exon 10 is skipped because of a distinct heterozygous 16 bp deletion (APC: c.1312 + 4_1312 + 19del) at the 5’ss of intron 10, and exon 9 is skipped because of a de novo indel (c.1226-1229delTTTTinsAAA) at the 5’ end of exon 9^141,142^. Exon skipping induces a frameshift, leading to the formation of premature stop codons. The resulting truncated protein lacks all functional domains and possesses only the homodimerization domain of wild-type APC, preventing the interaction of the mutant with other proteins such as β-catenin or axin. Therefore, β-catenin levels are increased, and the expression of growth-promoting genes can be increased through downstream T-cell transcription factor (Tcf) pathways. Mutations inducing exons 12 and 13 skipping resulted in the loss of armadillo functional domains without disrupting the open reading frame, and patients carrying this variant showed a less severe FAP phenotype than patients with truncation mutations^143^.

The LRP5 splice site mutation (NM_002335.4: c.686 + 1 G > T) identified in patients with familial exudative vitreoretinopathy (FEVR) causes LRP5 gene downregulation, resulting in complete retinal detachment and bilateral blindness and decreased BMD (bone mineral density)^144^. Several mutations that interfere with normal LRP5 splicing have been reported in OPPG (osteoporosis–pseudoglioma syndrome) patients, who also present with low BMD^145^. Gain-of-function mutations in the LRP5 gene leads to the acquisition of the high bone mass (HBM) phenotype in humans, whereas homozygous loss-of-function mutations can reduce the signaling activity of the Wnt signaling pathway, thereby reducing bone formation^146–148^. Mutations causing aberrant LRP5 splicing have also been shown to alter subcellular localization or disrupt intracellular transport compared to that of wild-type LRP5 and severely impair the Wnt pathway^145^.

DKK3 contains an alternative promoter that selectively carries exon 1a or 1b, which produces the same protein^149,150^. The exon 1a promoter showed stronger activity than the exon 1b promoter, and a 250 bp region further upstream of the promoter in exon 1a contained many CpG sequences. DKK3 expression downregulation has been associated with promoter methylation.

GSK3B shows an increased frequency with which exons 9 and 11 are skipped in the rs6438552 allele, which has been identified in PD (Parkinson’s disease) patients, and shows higher activity^151^. Additionally, five GSK3B splicing variants were identified in pig tissues, which showed differential expression patterns in fetal and adult tissues and were differentially regulated by insulin^152^. According to the GSK3β isoforms in pigs, the degree of influence on the mRNA expression of GY1 and GY2, encoding glycogen synthase, was different, and their roles in regulating phosphorylation and glycogen synthase activity were different. These findings suggest that GSK3β isoforms may play different roles in the insulin signaling pathway.

The homolog of the p53, p63, generates TAp63 and ΔNp63 isoforms due to an alternative promoter^153^. ΔNp63 lacking the N-terminal transactivation domain inhibits β-catenin phosphorylation and degradation mediated by GSK3β, resulting in β-catenin accumulation in the nucleus and increased transcriptional activity. Additionally, the TAp63 isoform exerts no effect, but the ΔNp63 isoform promotes normal mammary stem cell activation by enhancing Wnt signaling through increased Wnt receptor Fzd7 expression^154^. These findings suggest that ΔNp63 functions as a positive Wnt/β-catenin signaling regulator.

## Summary and conclusions

**Fig. 4:**
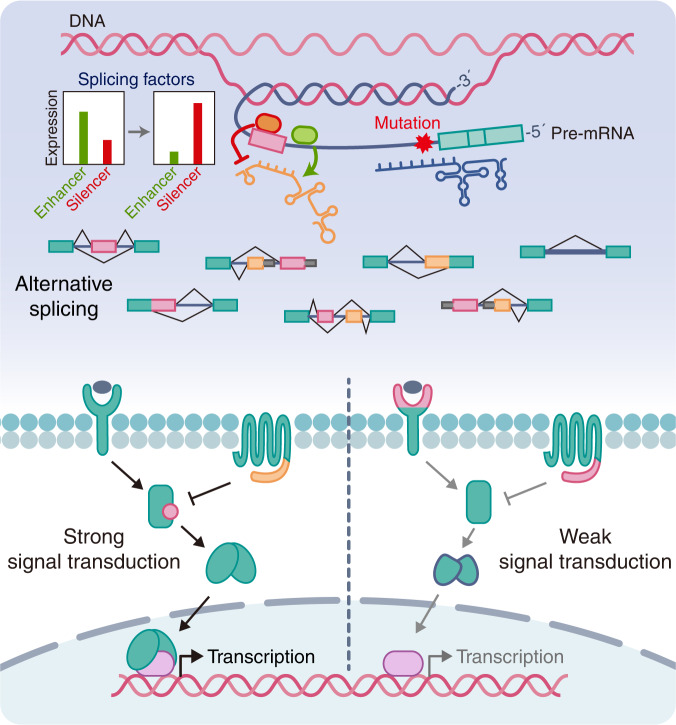
Roles of alternative splicing in cell signal transduction. Splicing factors systemically activate and regulate the alternative splicing of target gene pre-mRNA. A subset of genetic mutations induces aberrant alternative splicing. Alternative splicing produces multiple mRNAs, which increases proteome diversity. Splicing isoforms with differential functions induce the expression of specific gene sets by fine-tuning various signal transduction pathways. Therefore, dysregulation of alternative splicing leads to numerous human diseases.

Taken together, the studies presented herein show that alternative splicing is a very important mechanism that can enhance or attenuate signal transduction and has been shown to stably and precisely regulate various signal transduction pathways in living organisms (Fig. 4). PTMs, such as phosphorylation, are essential in regulating signal transduction, but because PTMs can also be affected by alternative splicing, the importance of alternative splicing needs to be further emphasized. Signal transduction can exert a synergistic effect while reinforcing signaling mediated through crosstalk among pathways. Examples include the effect of STAT3 activation on the Ras and PI3K/Akt pathways and the connections of JAK2 with the PI3K and ERK pathways^155–160^. Thus, the effects of splice variants may be broader than those identified in a single pathway.

With the advancement in sequencing technology, entire genome DNA sequencing and exome sequencing are being performed with patient samples. However, most of these studies are focused on mutation analysis that alters the amino acid sequence in exons. Although these mutations may create new splice sites or affect splicing regulatory elements, which may affect splicing, these aspects are overlooked. The mutation (c.2652 C > T; pV884V) located in JAK3 exon 19 does not change the amino acid sequence but activates a new 5’ss in exon 19, resulting in premature termination and no detectable protein expression^73^. Additionally, despite the functional importance of splicing isoforms, transcriptome analysis is largely focused on changes in total mRNA expression levels, and analysis based on splicing variants is rarely performed. However, in-depth studies on alternative splicing have reported that aberrant splicing events influence the onset and progression of various diseases. Short-read RNA sequencing, currently the most commonly used method of studying alternative splicing, is difficult to use to fully assemble or characterize complex isoforms due to the limited read length that can be accommodated^161–163^. Long-read RNA sequencing or third-generation sequencing, such as that enabled via an Iso-Seq platform developed by Pacific Bioscience and a direct RNA-seq platform developed by Oxford Nanopore Technology, shows great potential to overcome the issues of short-read RNA sequencing^164^. Compared to short-read RNA sequencing, long-read RNA sequencing increases mapping certainty, de novo assembly, isoform identification, and differential alternative splicing event detection. However, long-read transcriptomics is associated with high per-read costs, high error rates, and potential sequencing coverage bias; therefore, work is still needed to improve the accuracy of isoform expression quantification^161,165^. In addition to sequencing analysis, to distinguish between driver splicing, which is directly related to disease, and passenger splicing, which is not directly related to disease, the basic mechanism of alternative splicing and its functional role of resulting splicing isoforms should be further evaluated. These types of systematic studies will expand our understanding of pathogenesis associated with alternative splicing event and will help in developing targeted therapeutics.
